# Biocomposite of Cassava Starch-Cymbopogan Citratus Fibre: Mechanical, Thermal and Biodegradation Properties

**DOI:** 10.3390/polym14030514

**Published:** 2022-01-27

**Authors:** Zatil Hafila Kamaruddin, Ridhwan Jumaidin, Rushdan Ahmad Ilyas, Mohd Zulkefli Selamat, Roziela Hanim Alamjuri, Fahmi Asyadi Md Yusof

**Affiliations:** 1 Fakulti Kejuruteraan Mekanikal, Universiti Teknikal Malaysia Melaka, Durian Tunggal 76100, Melaka, Malaysia; zatilhafila@gmail.com (Z.H.K.); zulkeflis@utem.edu.my (M.Z.S.); 2 German-Malaysian Institute, Jalan Ilmiah Taman Universiti, Kajang 43000, Selangor, Malaysia; 3 Fakulti Teknologi Kejuruteraan Mekanikal dan Pembuatan, Universiti Teknikal Malaysia Melaka, Durian Tunggal 76100, Melaka, Malaysia; 4 School of Chemical and Energy Engineering, Faculty of Engineering, Universiti Teknologi Malaysia (UTM), Johor Bahru 81310, Johor, Malaysia; ahmadilyas@utm.my; 5 Centre for Advanced Composite Materials (CACM), Universiti Teknologi Malaysia (UTM), Johor Bahru 81310, Johor, Malaysia; 6 Faculty of Tropical Forestry, Universiti Malaysia Sabah, Jalan UMS, Kota Kinabalu 88400, Sabah, Malaysia; 7 Malaysian Institute of Chemical and Bioengineering Technology, Universiti Kuala Lumpur, Alor Gajah 78000, Melaka, Malaysia; fahmiasyadi@unikl.edu.my

**Keywords:** *Cymbopogan citratus* fibre, starch, natural fibre, biodegradation, mechanical properties

## Abstract

Increasing environmental awareness and concern have shifted the focus of research and development towards biodegradable materials development. In the current study, *Cymbopogan citratus* fibre (CCF) were incorporated into thermoplastic cassava starch (TPCS) with various content of CCF (10, 20, 30, 40, 50, 60 wt.%) via compression moulding. The determination of fundamental characteristics of TPCS/CCF biopolymer composites was conducted to assess their potential as biodegradable reinforcements. Characterization of the samples was conducted via Fourier-transform infrared spectroscopy (FT-IR), thermogravimetric analysis (TGA), and scanning electron microscopy (SEM), as well as mechanical, moisture absorption, and soil burial testings. The findings showed that the improved tensile and flexural features of the TPCS composites with CCF incorporation, with 50 wt.% CCF content yielded the maximum modulus and strength. The thermal properties of the biocomposite demonstrated that CCF addition improved the material’s thermal stability, as shown by a higher-onset decomposition temperature and ash content. Meanwhile, the CCF incorporation into TPCS slowed down the biodegradation of the composites. In term of morphological, homogeneous fibres and matrix dispersion with excellent adhesion was observed in morphological analyses using scanning electron microscopy (SEM), which is crucial for the enhancement of the mechanical performance of biocomposites.

## 1. Introduction

Plastics are incredibly durable and are widely used in industries such as packaging applications. Plastic production has been increasing over time due to the advantages of plastics, which have been making them a high-priority material in product manufacturing, such as, e.g., being lightweight, low cost, and convenient. However, plastic products created from petroleum-based polymers have detrimental effects on the environment by accumulating non-biodegradable waste, since they are entirely composed of chemicals such as ethylene and propylene [1]. As a result, biodegradable plastic is among the most promising alternatives to non-biodegradable plastics; consequently, interest in consuming available natural resources to manufacture more environmentally friendly polymers has been gradually rising to address the issue.

Nowadays, considerable effort is being made to develop natural based-biodegradable polymers with the goal of replacing petroleum-based polymers. In terms of performance and durability, these natural-based composites are regarded to be potential alternatives to the non-biodegradable fossils-based counterparts. Generally, renewable resources relate to plant-based elements, e.g., starch and cellulose [2]. Both of these materials are renewable and readily available from a variety of sources. Starch is an entirely biodegradable polysaccharide and the most promising material owing to its lower cost, wide availability, abundance, biodegradability, non-toxicity, and renewability [3,4,5]. Generally, starch is a crystalline material with two micro-sized structures—amylose and highly branched amylopectin [6]. When starch is exposed to a plasticizer and heat, spontaneous destructurisation occurs, which yields the thermoplastic starch [7]. However, compared to other plastics in use, pure thermoplastic starch has significant disadvantages, including high water absorption and poor mechanical properties, which limit its possible applications [8,9]. Therefore, thermoplastic starch modification is frequently required to prepare this material for use in real-world applications. To maintain its biodegradability, it appears that thermoplastic starch reinforcement with other natural fibres is a promising method. Recently, thermoplastic starch reinforcement with natural fibre has been proven a useful technique for addressing the limitations of thermoplastic starch [10]. Several studies have reported the incorporation of natural fibres, e.g., jute [11], wheat straw [2], coir [12], cotton [13], kapok [11], kenaf [14], corn [15], as well as sugar palm [10] into a thermoplastic starch matrix. Significant improvements were observed when natural fibres were used to reinforce starch-based materials, particularly the mechanical features, water barrier properties, and thermal stability [16]. These were mainly owed to the high structural similarity and correlation between cellulose and starch [2]. On the contrary, palm wax is also recognised as an excellent matrix because it has the potential to minimise the starch hydrophobicity, which in turn enhances the processability. Thermoplastic starch is prepared by using hot compression moulding of starch in the presence of plasticizer and fibre reinforcement. In our prior work, the addition of palm wax into thermoplastic starch resulted in biopolymer blends with improved properties [17]. Significant enhancements in the tensile modulus and strength of the TPCS was evidenced following the palm wax introduction into the TPCS, which was also ascribed to increasing the thermal stability. Therefore, the effect of *Cymbopogan citratus* fibre was additionally examined in this study using a TPCS/palm wax blend as the matrix.

Natural fibres have fascinated the interest of many due to their biodegradability, renewability, good adhesion of fibre-polar polymer matrix, low density, lightweight, non-corrosive nature, and easy availability [18,19]. Natural fibre reinforced composites have characteristics that are similar to traditional synthetic fibre reinforced composites. They have been considered as a substitute for fibres and inorganic fillers due to major environmental concerns. These naturally occurring polymers can be found in various forms, including leaves, stalks of plants, and grasses [20]. Natural fibres include cellulose, hemicellulose, and lignin, where hemicellulose and cellulose are both hydrophilic, while lignin is slightly hydrophobic [21]. Their energy efficiency, acceptable specific properties, environmental friendliness, and low production costs make natural fibres attractive reinforcement agents for polymer composites [22,23]. A preceding study reported by Prachayawarakorn et al. [16] on the development of silk protein fibre-thermoplastic rice starch found improved tensile strength and modulus and reduced elongation at break with a higher fibre content.

*Cymbopogan citratus,* or lemongrass, is among the aromatic members of the Poaceae family. This natural plant is cultivated in the tropical and semitropical countries of Asia, India, South America, and Africa [24]. *Cymbopogan citratus* fibre is gaining popularity in Malaysia as a natural material that can aid in the development of environmentally friendly resources. The plants typically grow up to 2.5 m × 1.2 m in height and width. *Cymbopogan citratus* has short rhizomes, and the leaves sprout directly from the soil [24]. *Cymbopogan citratus* plants are easy to grow and therefore produce a plentiful supply of all parts of the plants, including the leaves. The leaves can grow to 50 and 1.5 cm in length and width, respectively, with a greenish inside and glabrous. *Cymbopogan citratus* is a lignocellulose biomass containing approximately 37.5% cellulose, 11.4% lignin, and 22.2% hemicellulose [25]. In Malaysia, *Cymbopogn citratus* stalks are most commonly used as an ingredient to flavour foods in cooking, while the sharp leaves of the *Cymbopogn citratus* plant are discarded as waste. Due to the large amount of wastes produced, various waste reduction efforts have been made by avoiding disposal and repurposing them. In recent time, this plant fibre has been used for methylene blue dye removal by acting as an adsorbent from aqueous solutions, as a feedstock in pulp and paper preparation, and extensively used in pharmacological activities. Apart from that, *Cymbopogan citratus* fibre is among the alternative materials used as a reinforcing agent in polymer composites production [24,26,27]. 

Even though there are studies reported on using *Cymbopogan* species from China in synthetic polymers [28,29], none were found on the characterization of *Cymbopogan citratus* fibre from Malaysia in thermoplastic cassava starch matrix. Thus, the main objective of this research work is to study the effect of *Cymbopogan citratus* fibre originating from Malaysia on the mechanical, thermal, moisture, and biodegradation properties of TPCS. The ultimate goal of this study is to develop alternative biodegradable materials capable of reducing pollution and being more environmentally friendly.

## 2. Materials and Methods

### 2.1. Materials

**Figure 1:**
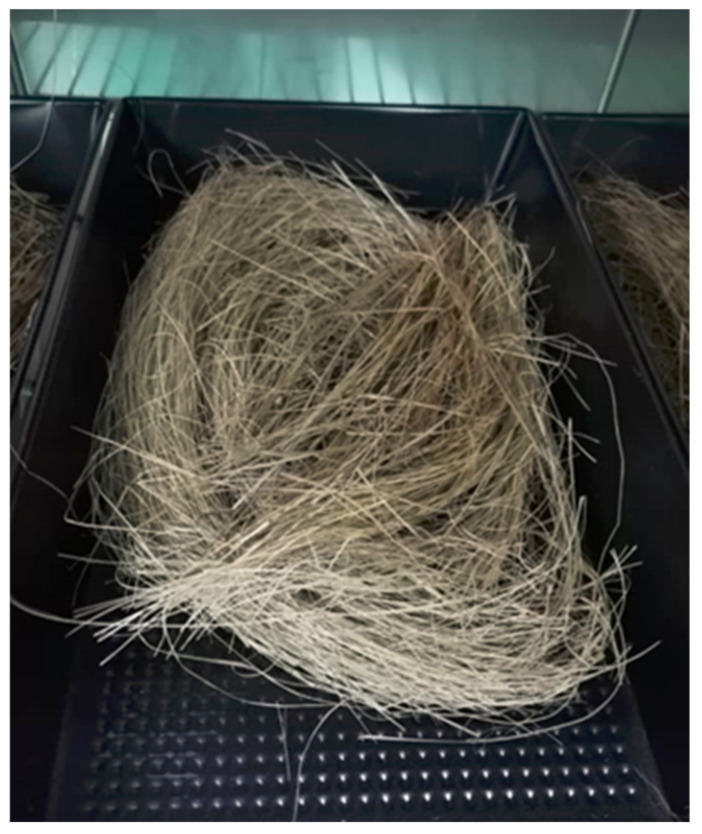
Fibre extraction from the *Cymbopogan citratus* plant.

The leaves of *Cymbopogan citratus* utilized in this research were obtained from the plants cultivated in Beranang, Selangor (West Malaysia). The *Cymbopogan citratus* fibre (CCF) were extracted via the water retting method by immersing the leaves in water for 28 days or 4 weeks (Figure 1). The leaves were then cleaned under running tap water, and manual removal of the fibres from the leaves was performed. The isolated fibres then underwent washing and drying for 5 h at 100 °C. Then, the dried fibres were cut into 1 cm and placed in a zip-locked plastic bag for additional processing. In this study, the cassava starch (food-grade) was purchased from Antik Sempurna Sdn. Bhd (Selangor, Malaysia). The analytical grade glycerol (99.5% purity) was procured from Qrec Chemicals Sdn. Bhd., (Selangor, Malaysia) and analytical grade refined palm wax was supplied by Green & Natural Industries Sdn. Bhd., (Selangor, Malaysia).

### 2.2. Sample Preparation

The thermoplastic cassava starch (TPCS) preparation was performed by mixing 65 wt.% starch, 30 wt.% glycerol, and 5 wt.% of palm wax. The mixture blending was conducted using a MX-GM1011 Dry Mixer from Panasonic (Shah Alam, Selangor, Malaysia) for 5 min at 1200 rpm at room temperature. The mixture was then hot-pressed at 150 °C for 30 min using a Technopress-40HC-B Plastic Hydraulic Moulding Press (Malaysia) under 10 tonnes load to yield plates with 3 mm thickness. The 30 min of hot-pressing was the minimum process time to make sure all of the remaining whitish starch powder was transformed into a transparent look (thermoplastic starch). TPCS/CCF composites were also fabricated using a similar method. The properties alteration of the matrix was carried out by incorporating various CCF ratios from 10 to 60 wt.%. Prior to the conditioning step, the prepared specimens were promptly placed in a silica gel-filled desiccator to avoid unpredicted moisture absorption.

### 2.3. FT-IR Analysis

The functional groups presence within TPCS and *Cymbopogan citratus* fibre was analysed using a Fourier transform infrared (FT-IR) spectroscopy. All samples’ spectra were generated from JASCO FTIR-6100 Spectrometer (Tokya, Japan), in the range of 4000–500 cm^−1^.

### 2.4. Scanning Electron Microscope (SEM)

The fractured tensile samples’ morphology was observed under a scanning electron microscope (SEM), model Zeiss Evo 18 Research, (Jena, Germany) at 10 kV acceleration voltage. Prior to the test, all samples were cut to a uniform size and gold-coated on the surface. After observation, all the specimens from the tensile test were kept in zip-locked plastic bags before characterization.

### 2.5. Differential Scanning Calorimetry (DSC) 

A differential scanning calorimeter (DSC) analysis of the TPCS reinforced with Cymbopogan citratus fibre were carried out using Universal V3-9A TA Tool, New Castle, PA, USA. During the procedure, each sample was weighed to a precision of 5 mg and put in an aluminium sample pan with a tightly sealed. The measurements were carried out in the temperature range 35–250 °C at a scanning rate of 10 °C/min, which was constant for the heating of all samples. Transfer temperatures were used to generate the thermogram values for the DSC cell, which was flushed with nitrogen gas to maintain an inert environment.

### 2.6. Thermogravimetric Analysis (TGA)

Thermo-gravimetric analyses were conducted employing a Mettler Toledo AG, Analytical (Schwerzenbach, Switzerland) from 25 to 600 °C, a constant heating rate (10 °C/min^−1^), and under a dynamic nitrogen atmosphere. Each sample, comprising 5–15 mg composite, was put in a sample pan and then heated. The generated TGA curve contained the weight loss percentage versus temperature.

### 2.7. X-ray Diffraction (XRD)

The crystallinity index (CI) of biocomposites were carried out using a Rigaku D/max 2500 X-ray powder diffractometer (Rigaku, Tokyo, Japan) employing *CuK*α radiation (λ = 1.5406 nm) at 35 mA and 40 kV, respectively. The crystallinity index of the samples CI (%) was calculated according to Equation (1):

(1) $$\mathrm{CI}=\frac{I002-Iam}{I002}\times 100\%.$$

### 2.8. Tensile Testing

The tensile properties of the samples were analysed following a standard procedure of ASTM D-638. The determinations of tensile strength and modulus, as well as elongation were performed in a Universal Testing Machine (INSTRON 5969), INSTRON (Noorwood, MA, USA) with a 50 kg load cell and constant crosshead speed of 5 mm/min. Measurements were carried out on five replications, and the tensile characteristics were computed as the mean of the measured values.

### 2.9. Flexural Testing

A Universal Testing Machine (INSTRON 5969), INSTRON (Noorwood, MA, USA) according to ASTM D-790 with a 2 mm/min crosshead speed and 50 kN load cell was used for the test. The samples of 130 mm (L) × 13 mm (W) × 3 mm (T) dimensions were used for five replications tested at 23 ± 1 °C temperature and of 50 ± 5% relative humidity.

### 2.10. Moisture Absorption

The TPCS/CCF biocomposites were put in a closed humidity chamber at 25 ± 2 °C temperature and relative humidity (RH) of 75 ± 2% to analyse the moisture absorption characteristics of the sample. Five samples of 10 mm × 10 mm × 3 mm dimensions were produced and oven-dried for 24 h at 105 °C ± 2 before the moisture absorption test. Weights were recorded prior to and after absorption, as indicated by *W_i_* and *W_f_* (Equation (2)), for a specified duration until a stable weight was attained. The moisture absorption value for all samples was computed following Equation (2):

(2) $$\mathrm{Moisture}\;\mathrm{Absorption}\;(\%)=\frac{W_{f}-W_{i}}{W_{i}}\times 100.$$

### 2.11. Soil Burial

The biodegradation test was performed following the method adopted from Jumaidin et al. [30]. The samples were frequently moistened using distilled water to maintain the humidity of the soil. The mean ambient temperature and relative humidity (RH) for the analysis were 26 ± 4 °C and 76 ± 4%, respectively, while the pH of the soil was 6.5. To summarise, an iron mesh was used for wrapping the samples before being buried in the soil to permit the elimination of degraded samples, while allowing moisture and microorganisms access. Before the testing, all samples underwent drying for a straight 24 h at 105 °C and the weights were measured and recorded to determine the initial weight, *M_i_.* All samples were soil-buried for prespecified durations of 2 and 4 weeks. Subsequently, the samples were carefully taken out of the soil at specific intervals and impurities were mildly cleansed using distilled water. Next, the degraded samples were oven dried for 24 h at 105 °C and reweighed to acquire the final weight, *M_f_*. The weight loss of the degraded samples was computed using Equation (3):

(3) $$\mathrm{Weight}\;\mathrm{loss}\;(\%)=\frac{M_{i}-M_{f}}{M_{i}}\times 100.$$

### 2.12. Statistical Analysis

Statistical analysis of the experimental findings was conducted via the analysis of variance (ANOVA) using SPSS software. The comparison of means was carried out using Duncan’s test at a significance level of 0.05 (*p* ≤ 0.05). 

## 3. Results

### 3.1. FT-IR Analysis

**Figure 2:**
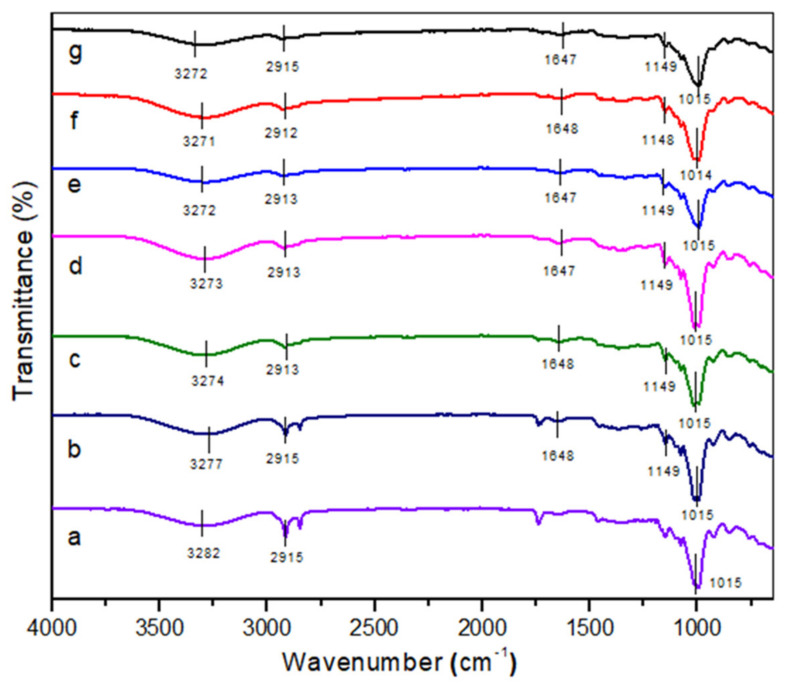
FT-IR spectra of (**a**) neat cassava starch matrix, (**b**) 10% CCF, (**c**) 20% CCF, (**d**) 30% CCF, (**e**) 40% CCF, (**f**) 50% CCF, (**g**) 60% CCF.

The modification of the TPCS matrix with *Cymbopogan citratus* fibre can be examined using the IR technique. Figure 2 presents the FT-IR findings for TPCS/*Cymbopogan citratus* fibre composites from 0 to 60 wt.% of fibre contents. Generally, all TPCS composites exhibited similar patterns, indicating that the TPCS is neither chemically altered nor changed by the *Cymbopogan citratus* fibre content in all composite samples. Parallel findings were reported from incorporating cotton and agar fibres into thermoplastic waxy rice starch [31]. Based on Figure 2, both pure TPCS and TPCS reinforced with *Cymbopogan citratus* fibre exhibited the same broad bands between 3200 to 3500 cm^−1^, indicating the existence of O–H groups. This phenomenon was caused by the hydroxyl groups contained in cellulose, hemicellulose, and lignin in the *Cymbopogan citratus* fibre [1]. This result also indicated that starch was highly water sensitive, owing to the existence of hydroxyl groups and stretching as a result of the hydrogen bonds holding the molecules [1,32]. Nevertheless, it was found that when the fibre content increased, the O–H bond peak changed to a smaller wavenumber. The O–H groups observed at 3300–3500 cm^−1^ exhibited a slight shifting to smaller wavenumbers linked to freely, intramolecularly, and intermolecularly bound hydroxyl groups [33]. Besides, the shifting in peak position also indicated that the composites possessed a good filler-matrix compatibility, where a lower wavenumber implied a stronger components interaction [34]. This shifting indicated the development of new hydrogen bonds between the components. Additionally, the generation of additional hydrogen bonds between the matrix and filler in the composite might be ascribed to an enhancement in the material’s mechanical properties, as hydrogen bonds provide greater deformation resistance. A comparable O–H trend was reported in a recent work conducted by Prachayawarakorn et al. [13] on thermoplastic rice starch/cotton fibre and thermoplastic waxy rice starch/cotton fibre development. This study provides compelling evidence of the new hydrogen bonds formed between the TPCS matrix and fibre [1].

Meanwhile, the band approximately within the range 2850–3000 cm^−1^ might be related to the component stretching vibrations of cellulose and hemicellulose in *Cymbopogan citratus* fibre [35]. Additionally, the band also indicated the vibration of C–H stretching produced by CH_2_ and/or CH_3_ [31]. Besides that, the C–O group presence at approximately 1000–1300 cm^−1^ corresponded to the C–O group stretching in the fibre, which is commonly found in hemicellulose and cellulose [36]. Meanwhile, the vibration peak around 1500 cm^−1^ was attributed to the lignin’s benzene ring vibration [30]. On the contrary, the 990–1000 cm^−1^ peaks were assigned to the stretching of an hydroglucose ring, O-C [37].

### 3.2. Scanning Electron Microscope (SEM)

**Figure 3:**
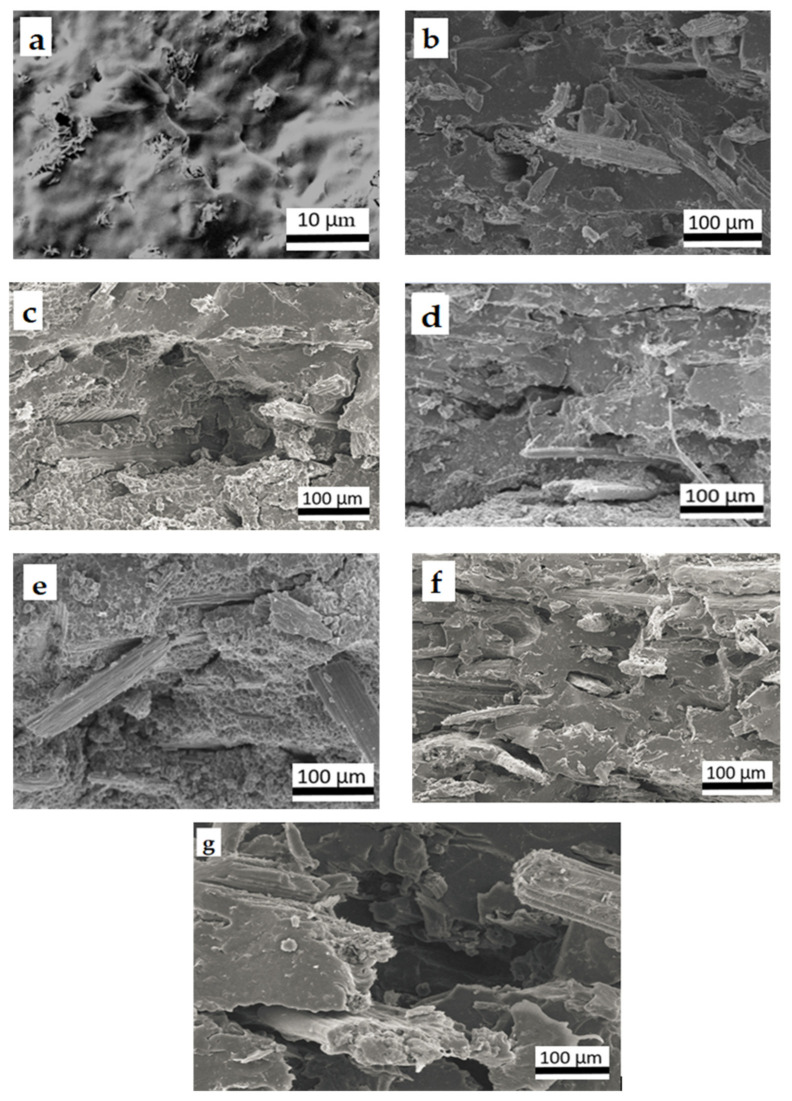
SEM micrograph of fractured TPCS surface blended with different ratios of *Cymbopogan citratus* fibre (**a**) TPCS, (**b**) TPCS/CCF-10 wt.%, (**c**) TPCS/CCF-20 wt.%, (**d**) TPCS/CCF-30 wt.%. (**e**) TPCS/CCF-40 wt.%, (**f**) TPCS/CCF-50 wt.%, (**g**) TPCS/CCF-60 wt.%.

The surface morphology of TPCS/CCF composites was also studied via SEM, and the fractured surfaces micrographs of the TPCS composites with varying ratios of *Cymbopogan citratus* fibre are presented in Figure 3. The cross-section images of samples showed microstructure variations in all specimens with different fibre contents. The neat TPCS sample showed a compact and a homogeneous surface without *Cymbopogan citratus* fibre reinforcement was found, as displayed in Figure 3a. This result was obtained from dry mixing cassava starch with glycerol to enhance cassava starch plasticization. Additionally, it was noticed that TPCS and CCF were highly compatible, as represented by the good fibre wetting of the matrices (Figure 3b). This effect could be explained by the identical hydrophilic properties of TPCS and CCF, which result in good adhesion between them. Meanwhile, Figure 3b–g presents the fibre breakage in all composites resulting from the tensile fracture, owing to effective stress transfer from the TPCS matrix to CCF, which contributed to the reinforcing effect of the composites. This discovery was in line with the results of tensile tests, which demonstrated increased tensile modulus and strength, as presented in tensile strength result. Despite this observation, the micrograph clearly shows fibre pull-out, and in particular that CCF was more broken (Figure 3d). This observation could be explained by TPCS and CCF, forming strong intermolecular hydrogen bonds [30]. Hence, composites with a higher CCF content appeared to have a good stress transfer from CCF fibre to the TPCS matrix and thus, subsequently enhanced the mechanical performance of the materials. 

Meanwhile, the surface morphology at a higher content showed the uneven distribution of the TPCS matrix and fibre. Figure 3g demonstrated that a higher amount of CCF (TPCS/CCF-60 wt.%) used in composites declined the tensile strength. The structure of the sample was less consistent, as indicated by the presence of more voids and CCF agglomeration on the fracture surface, owing to the high volume of fibre used, which has a detrimental effect on structural integrity [9]. Moreover, the declination of tensile strength might be associated with a higher fibre content, which was unable to properly mix with the matrix, resulting in inadequate dispersion with a rising fibre content. The matrix discontinuity effect was observed at high filler content, which led to insufficient matrix–fibre interactions and inadequate interfacial adhesion [38]. Other researchers have reported similar observations in this area on the development of thermoplastic starch [1].

### 3.3. Glass Transition Temperature (T_g_)

The TPCS/CCF composite was also analysed using differential scanning calorimetric study. The glass transition temperature, T_g_, is an extremely essential parameter in this study since it provides better understanding of the structure as well as the interaction between the TPCS matrix and fibre. T_g_ is defined as the glass transitions to rubber temperature and was normally estimated as the temperature at which the inflection point of the specific heat increment occurs at the glass-rubber transition [3]. The T_g_ values for all of the TPCS/CCF biocomposites were attained from the results of differential scanning calorimetric (DSC) analysis, as presented in Table 1. Besides, the inclusion of *Cymbopogan citratus* fibre from 0 to 60 wt.% yielded an increment in the T_g_ from 81.5 to 137.7 °C. This might be attributed to several reasons, firstly, the T_g_ increase that relied on the content of fibre might be attributed to the mobility restriction of segments of polymer molecules in the vicinity of fibres [39]. Secondly, the glass transition temperature (T_g_) shift indicates molecular interaction between the fibres and the matrix, which could be associated to the fibre–matrix bonding [40]. In other words, improved interfacial adhesion is achieved for the composites.

### 3.4. Thermogravimetric Analysis (TGA)

**Figure 4:**
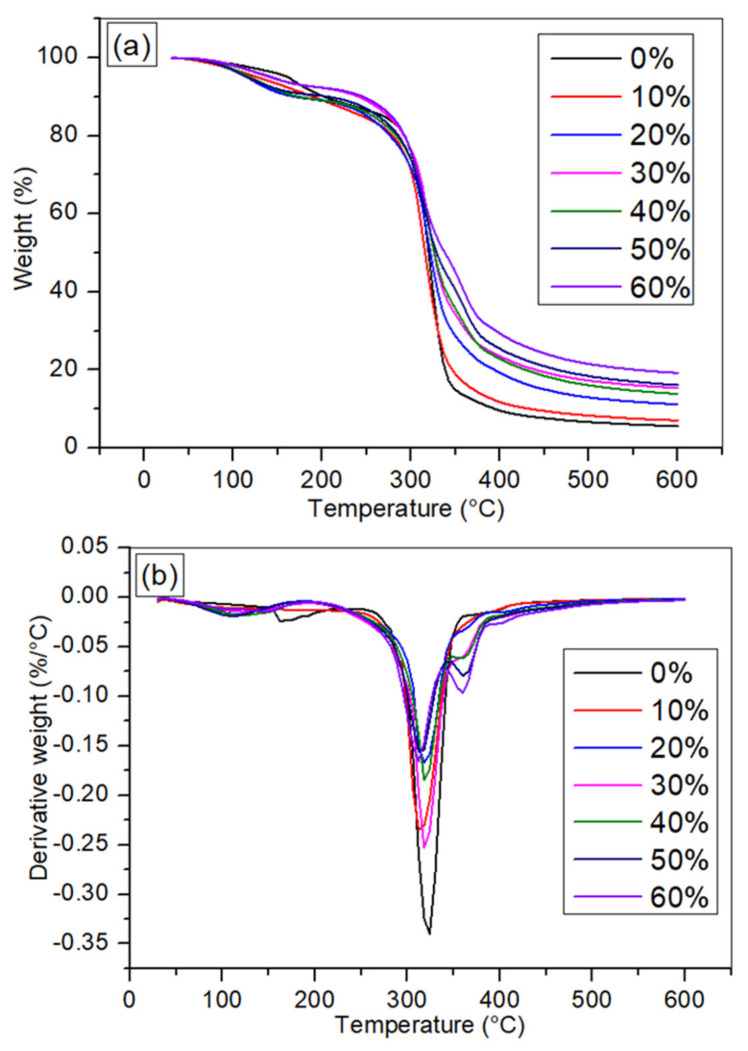
TGA findings of TPCS/CCF composites (**a**) TG curve (**b**) DTG curve.

Thermal decomposition of a thermoplastic starch (TPS)-based matrix is a critical study, since it identifies the processing, treatment, and operating limits of the materials. The mass loss, owing to the volatilisation of degraded products, is measured as a function of temperature in this analysis. Figure 4a,b presents the thermal stability and decomposition temperature of TPCS reinforced with *Cymbopogan citratus* fibre from 0 to 60 wt.% fibre contents. As the temperature is raised, the TGA thermograms show the weight reduction of the sample due to degradation. 

The first phased degradation that occurred was detected in the range 31–180 °C with 3.6–9.2% mass loss from the moisture evaporation of the water present in the materials. This was similar to the findings by Prachayawarakorn et al. [11] on thermoplastic cassava starch composites. Next, the further degradation between 150 to 380 °C for TPCS reinforced with *Cymbopogan citratus* fibre was ascribed to the three main natural fibre components decomposition: namely hemicellulose, cellulose, and lignin [41]. The process started with the degradation of hemicellulose, followed by cellulose, and then by lignin. Ash was yielded at the end of the process [42]. In this study, the hemicellulose and cellulose decompositions occurred at 200 °C, and their decompositions were completed at 270 °C [33]. Meanwhile, the lignin and the final cellulose decomposition took place from 270 to 370 °C [33]. This is corroborated by Reddy et al. [43], who determined the critical temperature for crystalline cellulose decomposition of 320 °C. Lignin degradation took a broad range of temperature, as early as 160 °C and up to 900 °C, with a smaller amount of weight losses [44]. On the contrary, the maximum decomposition of starch at approximately 300 °C could be due to the starch degradation accompanied by the fibre [11]. The ash formation took place when the degradation was completed due to the higher temperatures required for subsequent decomposition [45]. These findings corroborate previous research on the thermal behaviour of the composite prepared from thermoplastic cassava starch and reinforced banana leaf fibre [1].

Apparently, the addition of *Cymbopogan citratus* fibres into the TPCS matrix resulted in a few modifications of the thermal decomposition behaviour of the TPCS/CCF bio-composites. As shown by a comparison of obtained parameter values for the matrix and composites, *Cymbopogan citratus* fibres in the thermoplastic cassava starch matrix have a significant impact on mass loss of the composites. It was found that the mass loss decreased with rising fibre loading from 0 to 60 wt.% (Table 1), indicating the improved thermal stability of the samples. This result was consistent with the previous finding on the composite preparation from cassava starch reinforced with green coconut fibre, which revealed a gradual weight loss decrement with a rising fibre content and increased degradation temperatures when fibres were present in the prepared composites [39]. These were mainly ascribed to the high compatibility between starch and fibre as well as the higher thermal stability of the fibre compared to the starch. Additionally, when fibre was added, the excellent interaction between it and the matrix increased the thermal resistance of pure TPCS.

Meanwhile, the DTG curve for *Cymbopogan citratus* fibres reinforced TPCS-based composites (0 to 60 wt.%) are presented in Figure 4b. The results demonstrated DTG peaks placed between 300 and 400 °C with the highest peak at 0% matrix. However, when fibre was added, the DTG peaks reduced and showed minor changes [10]. From the DTG curve, the initial peak in the composites’ DTG curves (maximum decomposition) moved to a lower temperature with the presence of *Cymbopogan citratus* fibre, which was consistent with the findings found in the TG curve. Moreover, it was found from the DTG curve of the TPCS/CCF composites that three endothermic processes occurred. The first DTG peak was related to moisture elimination, while the fibre components decomposition began at the second stage, which resulted in the main DTG peak. The primary peak reflected the cellulose degradation, whereas the shoulder peak and the tail peak represented the hemicellulose and lignin degradations, respectively [46]. The final thermal event at 600 °C denoted the char residue (%) of the samples. 

Char is the residue material that remains after all volatile components in a material have been pyrolysed [47]. The content of char residue in TPCS/CCF composites was found to rise with the incorporation of *Cymbopogan citratus* fibre, where 60 wt.% fibre composites demonstrated the highest amount. This finding is consistent with the TG curve’s observation of an increase in the composites’ thermal stability above 300 °C. Improvement in the thermal stability of the composites compared to TPCS could be associated with the high carbonate composition of the fibre, which corresponded well with the results of the thermal degradation of the composites [48,49]. Thus, incorporating *Cymbopogan citratus* fibre from 0 to 60 wt.% has improved the rate of degradation of the composites, signifying *Cymbopogan citratus* fibre as being primarily accountable for the degradation results of the composites. Table 1 summarises the TGA result.

### 3.5. X-ray Diffraction (XRD)

**Figure 5:**
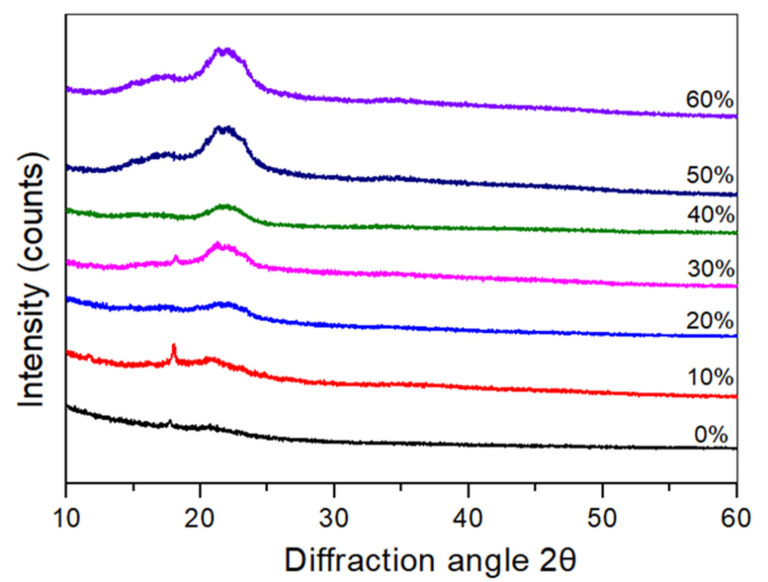
X-ray diffraction patterns of the TPCS/CCF composite.

The crystallinity of TPCS/CCF composite was determined using XRD to assess the effect of *Cymbopogan citratus* fibre loading on the crystallinity of the samples. The consistent diffractograms of the TPCS matrix and TPCS/CCF composite are shown in Figure 5. As can be observed in Figure 5, the segmentation between peaks exhibited similar pattern behaviour to the TPCS matrix and the only difference was that the main peaks strength increased after fibre loading was applied from 10 to 60 wt.%. The results from the XRD diffractogram have shown that the crystalline structure of the TPCS matrix was raised by the CCF incorporation, represented by its clear and well-defined peaks and negligible amorphous regions. The TPCS matrix exhibit sharp diffraction peaks at 2θ = 17.1°, indicating a typical A-type pattern [50]. Hence, it was anticipated that increasing the concentration of CCF in the samples would increase their relative crystallinity. The formation of crystalline structure of TPCS matrix samples without cellulosic fibre took place from the retrogradation or starch molecules recrystallisation as affected by heating. Retrogradation occurs when the amylose and amylopectin starch chains in the gelatinised paste is cooled after heating, resulting in the formation of molecular interactions of a more ordered crystalline structure [4].

Apparently, different phenomena are shown by TPCS/CCF composites samples. The relative crystallinity of the TPCS matrix was 18.3%. The reinforcement of CCF to the samples yielded an increase in the relative crystallinity up to 39.1% with 60% *Cymbopogan citratus* fibre content. This might be associated with the presence of cellulose fibre in TPCS matrix, which increased the main peaks intensity as well as improved the relative crystallinity of the TPCS/CCF composites [9]. A similar finding was noted in the cassava starch/green coconut fibre composites [39]. According to a previous study, increasing relative crystallinity was associated with an enhancement of tensile strength since the cellulose chain restricts the starch polymers movement, producing a brittle biocomposite [10]. This is corroborated by the fact that the strain value decreases as the cellulose content in the matrix increases. Consequently, increasing the amount of fibres in TPCS-based composites was projected to result in an increase in relative crystallinity, which was in line with the increasing tensile strength obtained in this experiment. Table 2 presents the relative crystallinity of TPCS matrix and TPCS/CCF composite.

### 3.6. Tensile Testing

**Figure 6:**
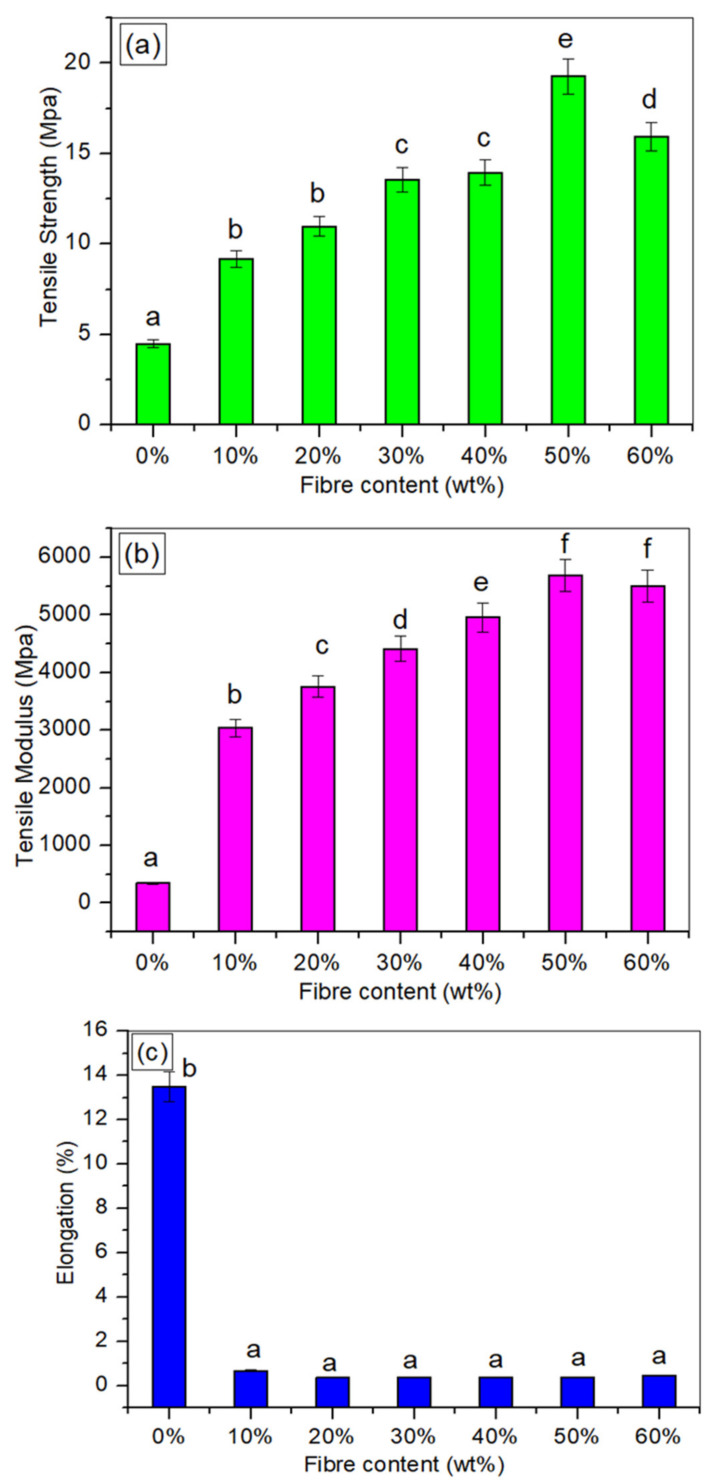
(**a**) Tensile strength, (**b**) tensile modulus, and (**c**) elongation at break (mm) for TPCS/CCF composites. * Values with different letters in the figures are significantly different (*p* ≤ 0.05).

The influence of *Cymbopogan citratus* fibre content on the mechanical features of TPCS/CCF composites was demonstrated in Figure 6. Table 2 presents the analysis of variance (ANOVA) findings of the tensile characteristics. The *p*-value (*p* < 0.05) indicated statistically significant variations in the mean values between the composite levels. The tensile test was primarily used to evaluate (a) tensile strength, (b) tensile modulus, and (c) elongation at break, respectively. Generally, the biocomposites demonstrated simultaneous increments in tensile strength and Young’s modulus, up to maximum values of 19.27 MPa and 5687.71 MPa, respectively, for CCF contents up to 50% wt. The tensile strength demonstrated a 331% increment (*p* < 0.05) and the tensile modulus was increased by 1546% (*p* < 0.05) (from 4.47 to 345.42 Mpa for neat TPCS) when the CCF loading reached 50 wt.%. It was obvious that the inclusion of CCF strengthened the tensile strength of the composites, and the optimal content of CCF reinforcement required was 50 wt.%. This behavioural increment in the tensile strength of the composites and modulus could be ascribed with a number of factors. Firstly, it is due to good adhesion between TPCS and CCF, which in turn provides an excellent matrix–fibre stress transfer [4,15]. This was demonstrated by the presence of fibre breakage on the tensile fracture surface, as shown in the SEM image. Secondly, the appearance of fibre fractures indicates excellent compatibility between the matrix and the fibre. This might be associated with the similarity in hydrophilicity of the TPCS matrix and the CCF fibre, thus leading to improved mechanical properties of the materials [32]. A parallel result was obtained in a work by Prachayawarakorn et al. [13] on the improvements in tensile modulus and strength of thermoplastic starch/cotton fibre composites. Thirdly, the behavioural enhancement in both the tensile modulus and strength of the composites might be associated with the chemical similarity between the CCF and TPCS matrix. This could be ascribed to the fact that the core cellulose structure, formed of hydroxyl functional groups, is comparable in both natural fibre and starch, resulting in high compatibility, specifically of the cellulose chains [51]. The tensile results were in line with preceding work on the mechanical characteristics of thermoplastic corn starch reinforced with corn husk fibre [15]. Another prior study on date palm stem fibres/epoxy composites revealed a similar result: the tensile modulus and strength increased with increased fibre loading [52].

However, a greater weight % of CCF content (60 wt.%) yielded in the decline of tensile modulus and strength. This could be attributed to higher fibre contents, which resulted in the tendency of the fibre to agglomerate in the starch matrix and being unable to distribute homogeneously between CCF and TPCS starch. This contributed to the poor mechanical properties [12]. Ayu et al. also reported that a high fibre content would result in improper wetting of the fibres by the matrix, fibre agglomeration, and blockage of the matrix stress transfer to the fibre [53]. Additionally, this phenomenon is explained by the matrix discontinuity’s effect on the matrix’s high fibre content, which led to a lack of matrix stress transfer to the filler [49]. This phenomenon can create poor matrix-to-fibres stress transfer and thus reduce the effectiveness as a reinforcing agent in TPCS. A prior study demonstrated a similar trend of results, with tensile strength and modulus increasing as the banana leaf fibre content increased from 10 to 40 wt.%, but the strength decreasing at 50 wt.% banana leaf fibre [1]. This finding was proved by the occurrence of some cracks on the matrix surface and agglomerate fibre, as seen in the SEM micrograph of the tensile fracture surface (Figure 3g).

Furthermore, the effect of fibre content on the elongation of the TPCS/CCF composites at break was inversely proportional to their tensile modulus and strength. The elongation of the TPCS/CCF biocomposites at break declined from 13.5 to 0.35% as the content of *Cymbopogan citratus* fibre was raised from 0 to 60 wt.% in the control TPCS composites. The incorporation of *Cymbopogan citratus* fibre content resulted in a decrement of the TPCS matrix’s molecular mobility, yielding stiffer biocomposite materials. As a result, the TPCS/CCF composites became stronger, stiffer, and less stretchy than the control TPCS sample [54]. Similar findings were found in previous work on the development of thermoplastic cassava starch following the introduction of jute and kapok fibres [11].

### 3.7. Flexural Testing

**Figure 7:**
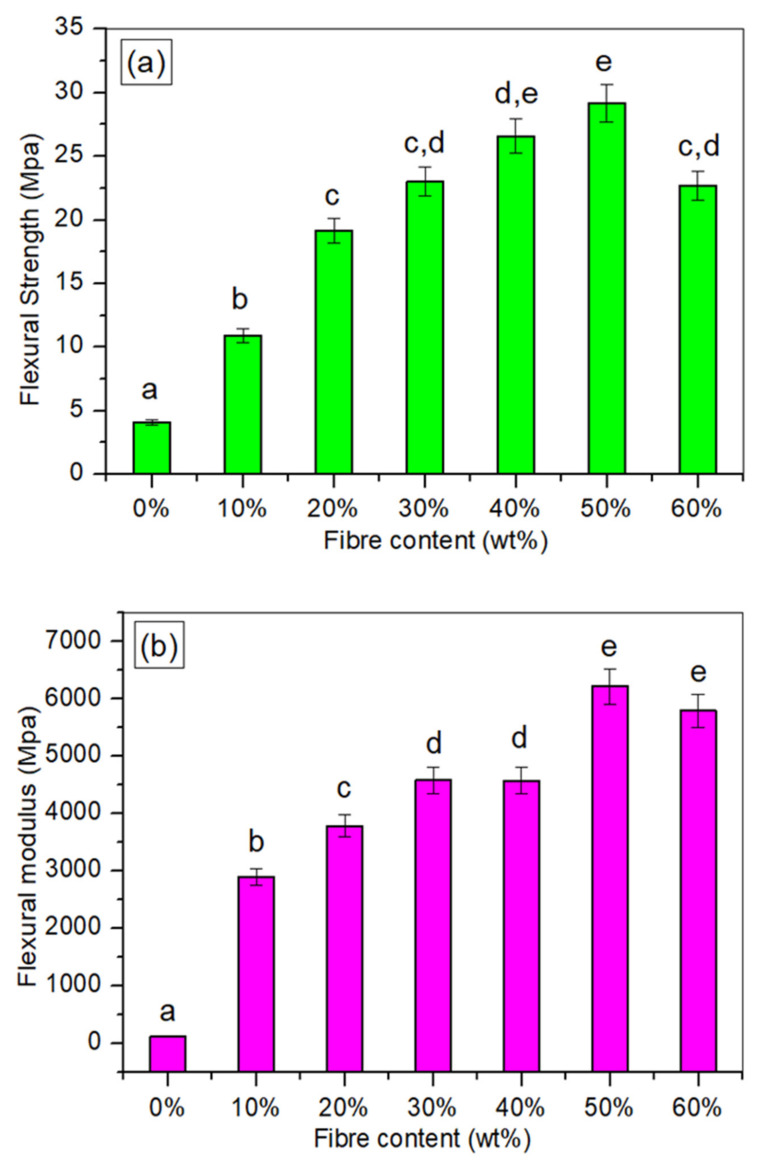
(**a**) Flexural strength and (**b**) flexural modulus for the TPCS/CCF composite. * Values with different letters in the figures are significantly different (*p* ≤ 0.05).

Figure 7a,b demonstrate the flexural strength and modulus of TPCS/CCF biocomposites, correspondingly. In general, the flexural characteristics of the composites exhibited a similar pattern to the tensile properties of the material, where 50 wt.% filler yielded a maximum flexural strength. The analysis of variance (ANOVA) of the flexural characteristics is presented in Table 3. A less than 0.05 *p*-value was obtained, indicating a statistically significant variation in the mean values of flexural strength and modulus between composite levels. Both the flexural strength and modulus were considerably improved (*p* < 0.05) as the *Cymbopogan citratus* fibre content was increased. The enhancement in the flexural characteristics of the composites might be due to the same factors as the tensile finding. The flexural strength increased by 609.48%, while the modulus increased by 5207.53% with the addition of 50 wt.% filler loading. This outcome was attributed to the fibre addition as reinforcement, which resulted in increased interfacial adhesion due to the presence of excellent stress transfer between the fibre and matrix [55]. This discovery was also verified by Ibrahim et al. [8], who incorporated date palm fibres into the thermoplastic starch matrix, which resulted in flexural strength and modulus increments. However, at 60 wt.% fibre weight, the flexural strength was reduced, which could be related to the poor fibre dispersion and non-uniformity of the interfacial bonding between the matrix and fibre [56]. These findings were in good agreement with the findings by Elsayed et al. [57] for incorporating flax fibre into thermoplastic starch composites. This study also revealed rises in flexural strength and modulus with rising filler content, with 50 wt.% flax fibres providing the highest flexural strength. Furthermore, the decrease in modulus at 60 wt.% fibre content may be due to an inadequate matrix amount to cover the entire *Cymbopogan citratus* fibre surface [1]. Vignesh et al. [58] noted a similar result, whereby they found that the flexural strength and modulus were raised significantly to 111.11 MPa with the incorporation of 50 wt.% fibre loadings. The biocomposite appeared less ductile when compared with the TPCS matrix, indicating that the fibre and matrix have a strong interfacial bond. 

### 3.8. Moisture Absorption

**Figure 8:**
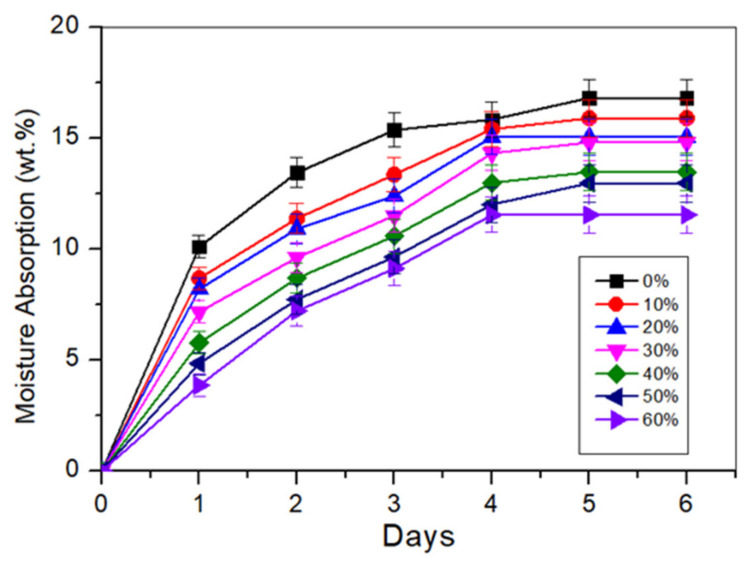
Moisture absorption curve of the TPCS/CCF composites.

Moisture absorption testing is a technique used to evaluate how much moisture a substance absorbs when exposed to a particular temperature and humidity. In addition, moisture sensitivity is an essential consideration for many starch-based products in various applications. Figure 8 presents the fibre loading effect on the moisture absorption behaviour of TPCS blends. It could be observed from the graph that moisture absorption was faster at the beginning of the storage period and became slower as the period extended. Previously published research on the moisture absorption of starch biopolymer matrix revealed similar findings [59]. In general, a steadier moisture absorption was observed after storing for 3 days, indicating that equilibrium moisture content was reached by the neat starch and composites with the surrounding environment. The moisture absorption often began to slow down and 17tabilize after 5–6 days.

As demonstrated in Figure 8, the introduction of *Cymbopogan citratus* fibre with TPCS yielded a great improvement in the water-resistance of the biopolymer composites than the neat TPCS matrix. For the TPCS/*Cymbopogan citratus* fibre composites, moisture absorption decreased as the fibre content increased. The neat TPCS biopolymer demonstrated a maximum moisture absorption percentage, followed by TPCS/CCF-10, TPCS/CCF-20, TPCS/CCF-30, TPCS/CCF-40, TPCS/CCF-50, and TPCS/CCF-60, with values of 16.82% to 12.90% respectively. This might be due to the higher hydropholicity of starch than cellulose, and was reduced with the existence of fibre [45]. Additionally, the chemical composition of *Cymbopogan citratus* fibres with lignin content as well as the presence of wax on their surface enhanced the moisture resistance of the composites [60]. Sahari et al. [61] reported a similar result when sugar palm fibre reinforced sugar palm starch with reinforcement of sugar palm fibres exhibited lower moisture absorption than the neat starch matrix. From the present study, it was concluded that the addition of CCF reduces moisture absorption in biocomposite, hence reducing the moisture sensitivity of this material. Additionally, this behaviour might be linked to increased interfacial interactions between the fibres and matrix, and the ability of the fibre to hinder absorption.

### 3.9. Biodegradation

**Figure 9:**
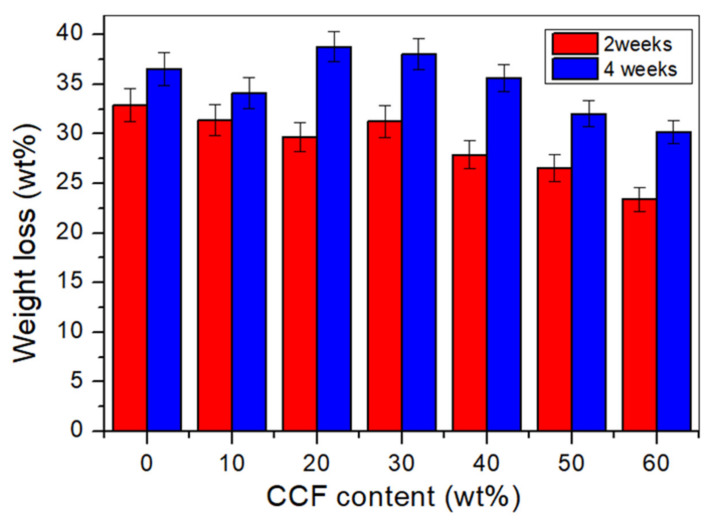
Weight loss of TPCS/CCF composites after soil burial for 2 and 4 weeks.

The material weight loss can be used to determine the rate of biodegradation by moisture and microorganisms throughout the soil burial period [62]. The weight loss percentage of thermoplastic cassava starch with the *Cymbopogan citratus* fibre addition after two and four weeks of soil burial is shown in Figure 9. Weight losses were greater in all composites after 4 weeks of burial rather than after 2 weeks. This result can be attributed to more microorganism activity during the prolonged burial time, increasing the weight loss of the material [33]. 

The addition of *Cymbopogan citratus* fibre into the TPCS matrix resulted in a smaller weight percentage change than the TPCS matrix. It can be observed that the TPCS matrix showed a high amount of weight loss from 32.8% and 36.5%, respectively, after 2 and 4 weeks of soil burial, while the TPCS/CCF-60 wt.% composite exhibited a lower weight percentage change than the TPCS matrix. This phenomenon could be due to the higher hydrophilicity of fibre than TPCS, which contributed to the lesser weight loss of the TPCS/*Cymbopogan citratus* fibre composites. TPCS with 60 wt.% *Cymbopogan citratus* fibre showed the smallest value, at 23.37% and 31.17% for 2 and 4 weeks, respectively. Additionally, the enhanced hygroscopic characteristics of the material may boost the microbes growth during degradation, hence boosting the weight loss of the material during breakdown [62]. Therefore, a lower weight loss of TPCS/*Cymbopogan citratus* fibre composites can be ascribed to greater resistance to biodegradation of CCF due to less hydrophilicity of the materials. This result widely validates the work of other researchers on the incorporation of silk protein fibre into thermoplastic rice starch [16].

## 4. Conclusions

Novel biocomposite produced from thermoplastic cassava starch reinforced by the different *Cymbopogan citratus* fibre concentrations was produced using hot pressing, and their mechanical, thermal, morphological, biodegradation, as well as moisture properties were investigated. The experimental result revealed new hydrogen bond formation between starch and *Cymbopogan citratus* fibre, as detected by the IR peak shift, which shows good compatibility between *Cymbopogan citratus* fibre and TPCS. The flexural and tensile characteristics of the composites revealed noticeable improvements with the addition of *Cymbopogan citratus* fibre. Additionally, the addition of *Cymbopogan citratus* fibre enhanced the thermal stability of the composite, as evidenced by the composites having a higher decomposition temperature than the pure TPCS. The tensile fracture morphological exhibited fibre breakage, indicating good dispersion of *Cymbopogan citratus* fibre within cassava starch and good adhesion between TPCS matrix and fibre. Samples with a higher *Cymbopogan citratus* fibre content at 60 wt.% showed a decrement in the mechanical properties. Incorporating *Cymbopogan citratus* fibre enhanced the physical properties of the composites, which were demonstrated by the decline in the moisture absorption capacity of the material. Nevertheless, the biodegradation rate of the material was determined using biodegradation testing, which revealed that the incorporation of *Cymbopogan citratus* fibre significantly reduced the biodegradation rate of the material. Overall, TPCS reinforced *Cymbopogan citratus* fibre composites have demonstrated potential as a replacement to the non-environmentally friendly polymers.

## Figures and Tables

**Table 1:** TGA results of TPCS/CCF composites.

Samples	T_g_ (°C)	T_on_ (°C)	T_max_ (°C)	Weight Loss at T_max_ (wt.%)	Char at 600 °C (wt.%)
TPCS	81.5	280	337	75.35	5.53
TPCS/CCF-10%	106.6	271	334	63.34	7.08
TPCS/CCF-20%	115.8	268	326	62.12	11.36
TPCS/CCF-30%	117.2	267	318	61.14	15.07
TPCS/CCF-40%	124.1	265	312	60.11	13.99
TPCS/CCF-50%	126.9	261	304	60.03	16.38
TPCS/CCF-60%	137.7	259	303	59.45	19.01

**Table 2:** Crystallinity index of the TPCS/CCF composites.

Samples	Crystallinity Index (%)
TPCS	18.3
TPCS/CCF-10%	19.1
TPCS/CCF-20%	23.4
TPCS/CCF-30%	24.7
TPCS/CCF-40%	31.7
TPCS/CCF-50%	37.7
TPCS/CCF-60%	39.1

**Table 3:** Analysis of variance (ANOVA) summary of TPCS/CCF composites.

Variables	df	Flexural Strength	Flexural Modulus	Tensile Strength	Tensile Modulus	Elongation at Break
Mixture	6	0.00 *	0.00 *	0.00 *	0.00 *	0.00 *

Note: * Significantly different at *p* < 0.05.

## Data Availability

The data presented in this study are available on request from the corresponding author.
